# The Influence of Comonomer on Ethylene/α-Olefin Copolymers Prepared Using [Bis(*N*-(3-*tert* butylsalicylidene)anilinato)] Titanium (IV) Dichloride Complex

**DOI:** 10.3390/molecules16021655

**Published:** 2011-02-15

**Authors:** Patcharaporn Kaivalchatchawal, Pattiya Suttipitakwong, Sutheerawat Samingprai, Piyasan Praserthdam, Bunjerd Jongsomjit

**Affiliations:** 1 Center of Excellence on Catalysis and Catalytic Reaction Engineering, Department of Chemical Engineering, Faculty of Engineering, Chulalongkorn University, Bangkok 10330, Thailand; 2 Innovation & Technology, PTT Chemical Public Company Limited, Tambon Map Ta Phut, Amphoe Mueang Rayong, Rayong 21150, Thailand

**Keywords:** post metallocene catalyst, homogeneous catalyst, FI catalyst, copolymerization

## Abstract

We describe the synthesis of [bis(*N*-(3-*tert-*butylsalicylidene)anilinato)] titanium (IV) dichloride (Ti-FI complex) and examine the effects of comonomer (feed concentration and type) on its catalytic performance and properties of the resulting polymers. Ethylene/1-hexene and ethylene/1-octene copolymers were prepared through copolymerization using Ti-FI catalyst, activated by MAO cocatalyst at 323 K and 50 psi ethylene pressure at various initial comonomer concentrations. The obtained copolymers were characterized by DSC, GPC and ^13^C-NMR. The results indicate that Ti-FI complex performs as a high potential catalyst, as evidenced by high activity and high molecular weight and uniform molecular weight distribution of its products. Nevertheless, the bulky structure of FI catalyst seems to hinder the insertion of α-olefin comonomer, contributing to the pretty low comonomer incorporation into the polymer chain. The catalytic activity was enhanced with the comonomer feed concentration, but the molecular weight and melting temperature decreased. By comparison both sets of catalytic systems, namely ethylene/1-hexene and ethylene/1-octene copolymerization, the first one afforded better activity by reason of easier insertion of short chain comonomer. Although 1-hexene copolymers also exhibited higher molecular weight than 1-octene, no significant difference in both melting temperature and crystallinity can be noticed between these comonomers.

## 1. Introduction

Nowadays, polymers and plastics, especially linear low-density polyethylene (LLDPE), are playing the important role on the material industry due to their low density, high strength and cost-effectiveness. It is generally known that a common means of generating LLDPE is the copolymerization of ethylene and α-olefins such as 1-butene, 1-hexene, 1-octene and 1-decene [1]. The catalytic systems used have an effect on the structure and properties of the synthesized copolymers, therefore, much effort has been directed towards the development of highly active olefin polymerization catalysts. Normally, Ziegler-Natta catalysts produce copolymers with a wide molecular weight distribution (MWD) and chemical composition distribution (CCD) as a result of their multiple active sites [2,3]. Conversely, homogeneous metallocenes and post metallocenes are single sites, leading to very uniform polymers with narrow MWD and CCD. Particularly, the FI complexes (Fujita group invented catalysts) bearing phenoxyimine ligands that have been fully developed by Fujita and coworkers may be regarded in the forefront of these developments and have gained much attention in both academia and industry as potential olefin polymerization catalysts. This is probably because of high activities comparable to or exceeding those of the group 4 metallocene catalysts and the uniform properties of the polymers produced [4]. 

Concerning linear low-density polyethylene, it is normally accepted that comonomers (both type and concentration) play an important role in the properties of the resulting copolymers in terms of melting behavior, density, crystallinity and mechanical properties [5,6]. For instance, the introduction of short-chain branching derived from the comonomer decreases the crystallinity and melting temperature of the copolymer. In other words, the comonomer contents, which in turn are sensitive to a large number of factors, namely the catalyst and cocatalyst structure and the initial comonomer concentration in the polymerization system, govern the copolymer melting behavior [7]. Furthermore, a number of recent studies have been revealed that the film performance, such as impact and tensile strength, increases with the comonomer length [6]. The size and concentration of comonomer units also affects the catalytic activities in homogeneous metallocene systems. Hence, in this present study, in order to investigate the influence of the initial comonomer concentration and the type of comonomer (1-hexene or 1-octene) on the catalytic performance of the catalyst and the copolymer properties, the synthesis of ethylene/α-olefins with Ti-FI catalysts have been performed using ethylene/comonomer in different proportions.

## 2. Results and Discussion 

### 2.1. Synthesis and characterization of titanium complex (Ti-FI catalyst)

**Scheme 1:**
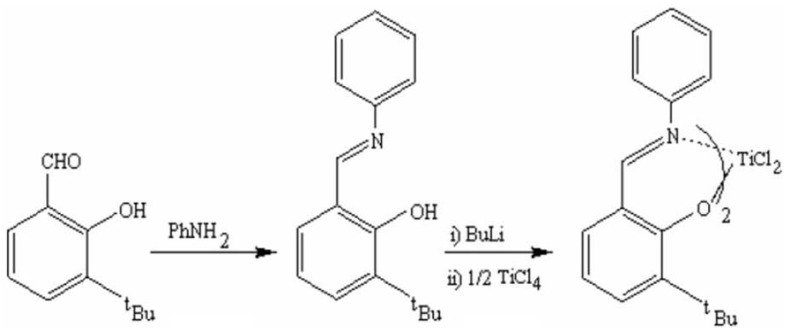
Synthetic route to the titanium complex.

The synthetic route for titanium complex is depicted in **Scheme 1**. 3-*t*-Butylsalicylaldehyde reacted with a primary amine via Schiff base condensation in ethanol to yield a *N-*(3-*tert*butylsalycilidene) aniline or phenoxyimine ligand. The titanium complex was obtained as reddish brown crystals by reaction of two equivalents of the lithium salt of the phenoxyimine ligand with TiCl_4_.

**Figure 1:**
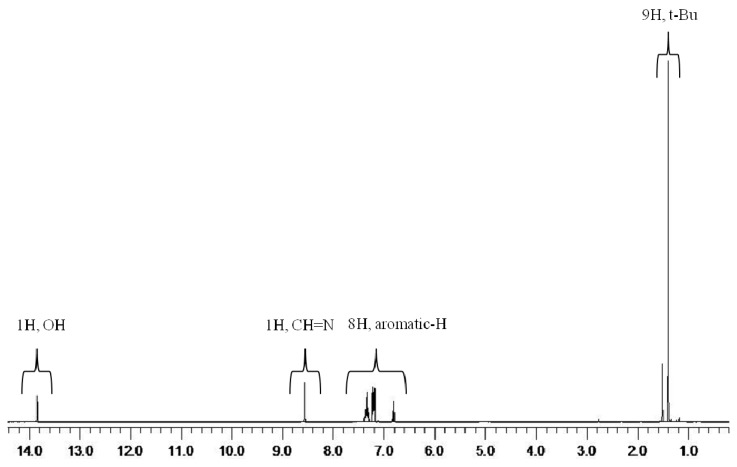
^1^H-NMR of *N*-(3-*tert-*butylsalicylidene) aniline or phenoxyimine ligand.

**Figure 2:**
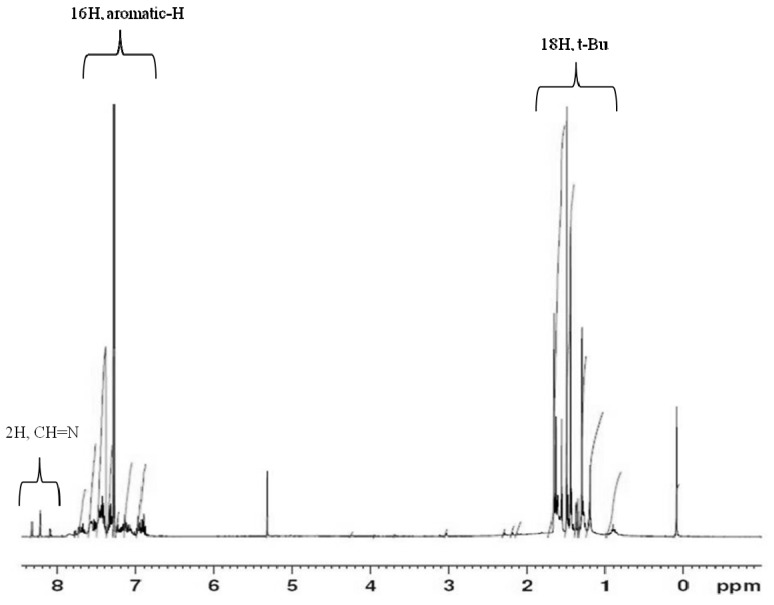
^1^H-NMR of Ti-FI complexes having phenoxyimine ligands.

The ^1^H-NMR spectra of the phenoxyimine chelate ligand *N*-(3-*tert*butylsalicylidene) aniline and Ti-FI complexes having phenoxyimine ligands are shown in **Figure 1** and **Figure 2****,** respectively. 

**Figure 3:**
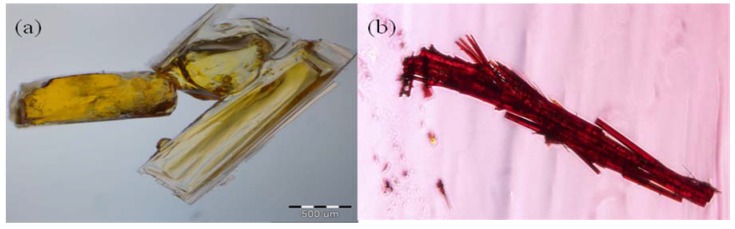
Optical microscope photographs of (a) Ti-FI ligand (b) Ti-FI complex.

In addition, using to the synthesis method mentioned, the Ti-FI ligand and complex can be crystallized into crystals and further characterized by optical microscopy to examine their morphologies, as shown in **Figure 3**. It can be seen from this figure that both of them exhibited the needle-like crystals of yellow and reddish brown color, respectively.

### 2.2. Catalytic properties of ethylene and ethylene/α-olefins copolymerization

The laboratory scale Ti-FI catalyst/MAO catalyzed ethylene/α-olefin copolymerization reaction was carried out at 323 K with constant ethylene pressure (50 psi). After filtration and drying, polymer was weighed and analyzed by ^13^C-NMR, GPC and DSC. The results are summarized in **Table 1**. 

**Figure 4:**
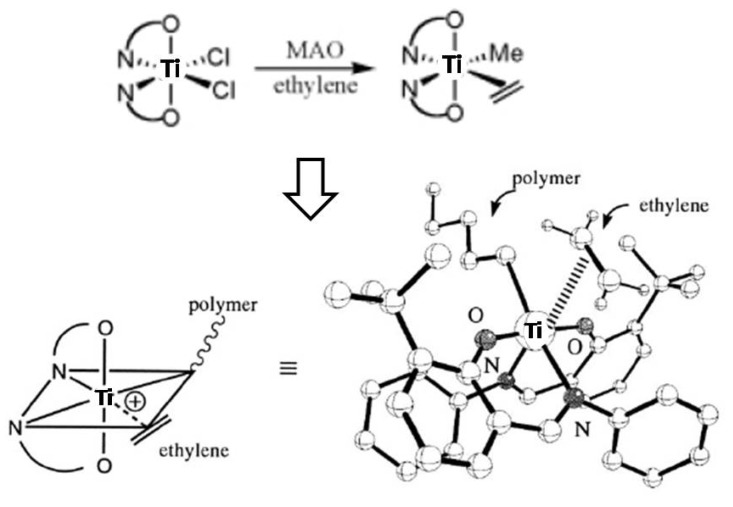
Structure and possible active species of titanium complex [8].

Concerning **Table 1**, the combination of Ti-FI catalyst with MAO in toluene solvent exhibited high activity (3,491 kg PE/mol Ti·h). To compare the catalytic activity of this catalyst with a metallocene catalyst, the commercial *rac*-Et[Ind]_2_ZrCl_2_ catalyst was used to produce polyethylene under the same polymerization conditions. It was found that the activity value exceeded the activity obtained with the commercial cyclopentadienyl ligand metallocene catalyst, indicating that phenoxyimine chelate ligands have good potential as olefin polymerization catalysts. This may be attributed to the fact that these ligands posses moderate electron-donating properties species as well as a pair of available *cis*-located sites for polymerization [2,8]. In order to obtain more detailed insights into the catalytic activity during the polymerization, the possible active species of the titanium complex as determined by Fujita *et al*. from DFT calculations and X-ray crystallographic analysis [4,8] and the ethylene polymerization mechanism can be represented in the **Figure 4**. The mechanism begins with the activation of the catalyst with an appropriate cocatalyst – MAO in this case – to form an alkyl cationic complex, having the two available *cis*-located sites needed for polymerization. As a result, two chlorine bound sites were turned into polymerization sites, in other words, a growing polymer chain site and an ethylene coordination site, leading to the propagation of growing polymer chain.

Moreover, it can be observed from **Table 1** that no polymer was obtained from the system that was composed of only 1-hexene or 1-octene as monomer (entry H4 or O4). This result corresponded to the finding of Chaichana *et al*. [5] who examined the catalytic behavior of constrained geometry catalyst (CGC). They reported that the system presented no catalytic activity for in 1-hexene polymerization. Conversely, entry E2, which performs the ethylene homopolymerization is capable of attaining high polymer yields, suggesting that TiC_34_H_36_Cl_2_N_2_O_2_ (Ti-FI) acts as a high potential catalyst for ethylene polymerization, despite its poor performance for 1-hexene or 1-octene homopolymerization. This may be ascribed to the fact that ethylene is the most active monomer; thus, it can insert into the active site and then form polymer chains by itself, whereas the other monomers, namely 1-hexene and 1-octene, are too large and less active; accordingly, the initiation polymerization step cannot occur because of a lack of space for insertion of a bulkier monomer. On the other hand, when the system contains both ethylene and a bulky comonomer like 1-hexene or 1-octene, it turns out that the initiation step can be accomplished by the displacement of ethylene. This step provides higher space for longer 1-alkene and after that the propagation can proceed into the growing chain of copolymer.

The comparison of catalytic activities of two sets of catalytic system, containing using 1-hexene (H1-H4) and 1-octene (O1-O4) as comonomers by using Ti-FI as catalyst can be obtained from **Table 1**. The activities of both copolymerization systems were higher than in ethylene homopolymerization, a feature generally known as the “comonomer effect”, which is a quite general effect of many comonomer pairs. Moreover, the catalytic activity increases with the increase of comonomer feed concentration. Several possible causes have been proposed to explain this behavior; however, the simplest and most supported is the improvement of monomer diffusion to the catalyst center on account of the crystallinity reduction in polymer structures when introducing a small amount of comonomer [7]. Another explanation for this phenomenon is that the inserted comonomer may resulted in the ion separation between the cationic active species and anionic cocatalyst providing more space for polymerization, thus enhancing the activity.

With regard to **Table 1**, the catalytic activity shows a general trend of 1-hexene > 1-octene under the same conditions with a fixed [ethylene]/[α-olefin] monomer feed ratio. Thereby, it is fair to say that the bigger size of 1-octene comonomer has a negative effect on ethylene/α-olefin copolymerization, which is probably due to more steric hindrance at the catalytic center.

### 2.3. Properties of polymers

The properties of the obtained homo and copolymers are gathered in **Table 2**. Comparing the PE obtained with both catalysts, it can be observed that the molecular weight of the homopolymer obtained with Ti-FI catalyst was considerably higher than that resulting from use of the metallocene catalyst. Besides, the commercial catalyst furnished quite high polydispersity when compared with the corresponding value with the FI catalyst. The uniform molecular weight distribution results were also seen in all the copolymers having 1-hexene and 1-octene units synthesized with Ti-FI catalyst, implying the single site polymerization mechanism of this catalyst. Another interesting point dealing with the molecular weight result is that the average molecular weight of the copolymer decreased with the increase of comonomer feed concentration. This might suggest that 1-hexene and 1-octene incorporation promotes chain termination reactions, consequently contributing to formation of lower molecular weight copolymers [9]. Furthermore, it can be seen from **Table 2** that the copolymers derived from using 1-octene as comonomer had a lower molecular weight than those obtained from 1-hexene.

Regarding the thermal properties, 1-hexene and 1-octene incorporation resulted in a noticeable reduction of the melting temperature and crystallinity of the copolymers, when compared to the homopolymer (entry E-2), but the type of comonomer does not seem to influence both properties. This was in accord with the Flory’s theory [1] that described the relationship between the melting behavior and comonomer of copolymerization system. However, this decrease was less pronounced in the case of higher comonomer feed concentration, suggesting that the bulky structure of the Ti-FI catalyst possibly reduces and limits the accessibility of the comonomer incorporation onto the active sites, especially for high comonomer concentrations, thus having a small effect on the properties.

### 2.4. Microstructure of the polymers

The microstructure of copolymers, including the incorporation of comonomer and the comonomer triad distribution, can be determined from ^13^C-NMR spectroscopy. Nonetheless, in this work there was no peak which indicated the comonomer branch in the backbone for all copolymers, presumably due to the presence of only a small amount of α-olefin insertion, contrary to the α-olefin incorporation ability of commercial *rac*-Et[Ind]_2_ZrCl_2_. Jongsomjit *et al*. found that this commercial complex possessed a high insertion ability for 1-hexene and 1-octene, at around 40 and 29%, respectively [10,11,12,13,14]. In addition, this problem might occur because of the ultra high molecular weight of the resulting copolymers and the too low sample preparation temperature. Thereby, other techniques, such as CRYSTAF, infrared spectroscopy and pyrolysis gas chromatography mass spectrometry, should be further applied to investigate the copolymer microstructure in this case. 

### 2.5. Morphology of polymers

**Figure 5:**
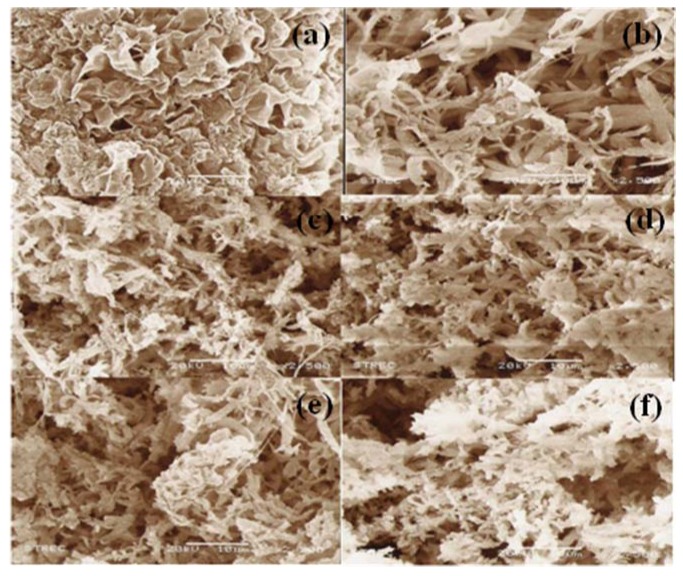
SEM photographs of polymers (a) E-1 (b) E-2 (c) H-1 (d) H-2 (e) H-3 (f) O-1.

The SEM micrographs of polymers are shown in **Figure 5**, indicating the typical morphologies of copolymers obtained from this catalytic system. There was no significant change in copolymer morphologies with the different comonomer lengths and initial comonomer concentrations employed. On the contrary, the morphology of polymers generated from Ti-FI catalyst seems to more rod-like comparing to those obtained from metallocene catalyst, suggesting that the catalyst microstructure might have an effect on the polymer morphology.

## 3. Experimental

### 3.1. Materials

All operations were handled under an argon atmosphere using glove box and/or standard Schlenk techniques. Ethylene (polymerization grade) was obtained from the National Petrochemical Co. Ltd., Thailand. 3-*tert*-Butylsalicylaldehyde, aniline and TiCl_4_ (99+%) were purchased from Aldrich Chemical Company, Inc., and used without further purification. Hexane, diethyl ether and dichloromethane (anhydrous grade) were purchased from Aldrich Chemical Company. Methylaluminoxane, (MAO, 10% in toluene) was donated BY PTT Research and Technology Institute (Thailand). Toluene was obtained by the Exxon Chemical, Thailand Co., Ltd. It was dried over dehydrated CaCl_2_ and distilled over sodium/benzophenone. Ultra-high purify (UHP) argon (99.999%) was purchased from Thai Industrial Gas Co., Ltd.. 1-Hexene and 1-octene (≥97%) was purchased from Aldrich Chemical Company, Thailand and further purified by distilling over CaH_2_ for 6 h. Commercial metallocene catalyst, rac-Et[Ind]_2_ZrCl_2_, was purchased from Aldrich Chemical Company (Thailand). ^1^H-NMR was used to investigate the structure of the ligand and catalyst complex. The spectra were recorded at ambient probe temperature (298K) using a Bruker AVANCE II 400 instrument operating at 400 MHz with an acquisition time of 1.5 s and a delay time of 4 s. Ligands and titanium complex solution were prepared using tetramethylsilane as solvent and deuterated chloroform for an internal lock. An Olympus BX51 instrument was employed to investigate the morphology of the ligand and the catalyst crystals by optical microscopy.

### 3.2. Titanium-FI complex synthesis

Bis[*N*-(3-*tert*-butylsalicylidene) anilinato]titanium (IV) dichloride (Ti-FI complex) was synthesized according to the procedure described by Saito *et al*. [15]. The synthesis of Ti-FI complex can be divided into two main steps, as follows:

#### 3.2.1. Synthesis of N-(3-tert-butylsalicylidene) aniline

Firstly, a stirred mixture of 3-*tert*-butylsalicylaldehyde (2.34 g, 13.4 mmol) and 3Å molecular sieves (2 g) in ethanol (20 mL), a solution of aniline (1.41 g, 15.1 mmol) in ethanol (10 mL) was added dropwise over a 1-min period at room temperature. Then, the mixture was stirred for 16 h and filtered. The 3Å molecular sieves were washed with ethyl acetate (20 mL). The combined organic filtrates were concentrated *in vacuo* to afford the crude imine compound. Finally, purification by column chromatography on silica gel using hexane/ethyl acetate (10:1) as eluent gave *N*-(3-*tert*-butylsalicylidene) aniline as orange crystals. ^1^H-NMR (CDCl_3_) δ = 1.45 (s, 9H, t-Bu), 6.81–7.35 (m, 8H, aromatic-H), 8.56 (s, 1H, CH=N), 13.84 (s, 1H, OH).

#### 3.2.2. Synthesis of Bis[N-(3-tert-butylsalicylidene)anilinato] titanium (IV) dichloride

To a stirring solution of N-(3-*tert*-butylsalicylidene) aniline (1.552 g, 6.13 mmol) in dried diethyl ether (50 mL) at 195 K, a 1.61 M hexane solution of *n*-butyllithium (3.80 mL, 6.08 mmol) was added dropwise over a 5-min period. The solution was allowed to warm to room temperature and stirred for 4 h. The resulting solution was added dropwise over a 30 min period to a stirred solution of TiCl_4_ (0.58 g, 3.06 mmol) in dried diethyl ether (90 mL) at 195 K. The mixture was allowed to warm to room temperature and stirred over night. After removal of the solvent, the product was extracted with CH_2_Cl_2_. Filtration following removal of the volatile gave a reddish brown solid. The solid was recrystallized from a dried dichloromethane/dried pentane (1:1) solution at room temperature to give bis[*N*-(3-*tert*-butylsalicylidene) anilinato] titanium (IV) dichloride. ^1^H-NMR (CDCl_3_): δ = 1.26–1.66 (m, 18H, t-Bu), 6.90–7.67 (m, 16H, aromatic-H), 8.22–8.33 (m, 2H, CH=N).

### 3.3. Polymerization procedure

The ethylene/α-olefin [(1-hexene, H) and (1-octene, O)] copolymerization reactions were carried out in a 100 mL semi-batch stainless steel autoclave reactor equipped with a magnetic stirrer. At first, the desired amounts of MAO ([Al]_MAO_/[Ti]_cat_ = 250) and the toluene were introduced into the reactor. The Ti-FI complex in toluene was put into the reactor to make the amount of catalyst 2.5 µmol. After that, the reactor was immersed in liquid nitrogen, followed by addition of the α-olefins into the frozen reactor. The reactor was heated up to the polymerization temperature at 323 K. By feeding a fixed amount of ethylene (0.018 mole ~ 6 psi) into the reaction mixture, the ethylene consumption can be observed corresponding to the ethylene pressure drop. Lastly, the reaction was terminated by adding acidic methanol. After filtration, the resulting polymers were washed with methanol and dried at room temperature.

### 3.4. Characterization of polymers

Differential scanning calorimetry: DSC thermal analysis was used to examine the thermal properties (T_m_ and heat of fusion) via a DSC 204 F1 Phoenix®. The DSC measurements were recorded during the second heating/cooling cycle with the heating rate of 10 °C/min during the range of temperature 30–200 °C.

Gel Permeation Chromatography: A high temperature GPC (Waters 2200) equipped with a viscometric detector, differential optical refractometer and four Styragel HT type columns (HT3, HT4, HT5 and HT6) was used to determine the molecular weight (MW) and molecular weight distribution (MWD) of polymer. The measurement was taken at 135 °C using 1,2,4-trichlorobenzene as a solvent and a mobile phase of 1 mL/min flow rate

Scanning electron microscopy: SEM was used to determine the sample morphologies using a JEOL mode JSM-5800LV instrument.

Nuclear magnetic resonance spectroscopy: ^13^C-NMR spectroscopy was used to determine the α-olefin incorporation and copolymer microstructure. Comparison of the positions of peak in the ^13^C- NMR spectra of polymer sample with characteristic leads to identification of the sequence of the comonomer incorporation, referring to Randall [16]. The spectra were recorded at room temperature using a Bruker AVANCE II 400 instrument operating at 100 MHz with an acquisition time of 1.5 s and a delay time of 4 s. The samples were prepared from using 1,2,4-trichlorobenzene as a solvent and deuterated chloroform for an internal lock.

## 4. Conclusions

This article has reported the preparation of LLDPE copolymer from ethylene/α-olefin copolymerization using TiC_34_H_36_Cl_2_N_2_O_2_ (Ti-FI catalyst) by varying the initial comonomer concentration and the comonomer type. The results reveal that all copolymers achieved very high activity, high molecular weight and narrow molecular weight distribution; on the other hand, the bulkiness of the catalyst structure is likely the main feature limiting the incorporation of comonomer into the polymer backbone. The increase of initial comonomer concentration caused an increase in catalytic activity; conversely, it led to a decrease in molecular weight and melting temperature. In addition, concerning the impact of the comonomer type on the catalytic behavior, 1-hexene exhibited apparently higher activity and molecular weight with corresponding 1-octene based copolymer. However, both melting temperature and crystallinity appeared to be independent of comonomer type. 

## Figures and Tables

**Table 1:** Summary of ethylene/α-olefins copolymerization catalytic activities.

Entry^a^	Ethylene^b^	Comonomer	Time	Weight	Activity^c^
	[mol/L]	[mol/L]	[s]	[g]	[kg polymer/mol Ti·h]
E-1	0.6	0	146.4	0.2881	2833
E-2	0.6	0	133.2	0.3230	3491
H-1	0.6	0.3	91.2	0.3496	5520
H-2	0.6	0.45	91.8	0.3647	5720
H-3	0.6	0.6	83.4	0.3766	6502
H-4	0	0.6	n.a.^d^	n.a.^d^	n.a.^d^
O-1	0.6	0.3	180	0.4405	3524
O-2	0.6	0.45	135.6	0.3748	3980
O-3	0.6	0.6	142.2	0.4470	4526
O-4	0	0.6	n.a.^d^	n.a.^d^	n.a.^d^

^a^ run E1 used commercial *rac*-Et[Ind]_2_ZrCl_2_ as catalyst; runs E2, H1-H4 and O1-O4 used Ti-FI as catalyst; comonomer of runs H1-H4 is 1-hexene; comonomer of runs O1-O4 is 1-octene; ^b^ Ethylene addition into the system; ^c^ Polymerization condition: [Ti] = 2.5 µmol, MAO as cocatalyst, [Al]/[Ti] = 250, liquid volume (toluene) = 30 mL, temperature = 323 K, ethylene pressure = 349 kPa (50 psi); ^d^ n.a. means not applicable (no observed polymer)

**Table 2:** Properties of resulting polymers.

Entry^a^	Feed ratio [E]/[α-olefin] [mol/mol]	Melting point^c^ (T_m_)[°C]	Crystallinity^d^ (X_c_)[%]	Molecular weight^e^ (M_w_)[kg/mol]	PDI^e^ (M_w_/M_n_)
E-1	1/0	134.5	38.78	432	2.5
E-2	1/0	134.3	64.01	920	1.60
H-1	1/0.5	128.7	41.94	729	1.66
H-2	1/0.75	126.3	44.93	768	1.69
H-3	1/1	127.0	43.21	675	1.67
H-4	0/1	n.a.^b^	n.a.^b^	n.a.^b^	n.a.^b^
O-1	1/0.5	128.0	42.21	668	1.64
O-2	1/0.75	127.2	48.13	641	1.63
O-3	1/1	128.3	50.37	633	1.65
O-4	0/1	n.a.^b^	n.a.^b^	n.a.^b^	n.a.^b^

^a^ run E1 used commercial *rac*-Et[Ind]_2_ZrCl_2_ as catalyst; runs E2, H1-H4 and O1-O4 used Ti-FI as catalyst; comonomer of runs H1-H4 is 1-hexene; comonomer of runs O1-O4 is 1-octene; ^b^ n.a. means no observed polymer; ^c^ determined by DSC; ^d^ estimated by dividing the heat of fusion of polymer (measured by DSC) by 294 J/g of the theoretical value for 100% crystalline PE homopolymer; ^e^ determined by GPC
